# The Role of the C-Clamp in Wnt-Related Colorectal Cancers

**DOI:** 10.3390/cancers8080074

**Published:** 2016-08-03

**Authors:** Aditi J. Ravindranath, Ken M. Cadigan

**Affiliations:** Department of Molecular, Cellular and Developmental Biology, University of Michigan, Ann Arbor, MI 48109, USA; aditir@umich.edu

**Keywords:** TCF/LEF, C-clamp, Wnt, β-catenin, colorectal cancer

## Abstract

T-cell Factor/Lymphoid Enhancer Factor (TCF/LEF) transcription factors are major regulators of Wnt targets, and the products of the *TCF7* and *TCF7L2* genes have both been implicated in the progression of colorectal cancer in animal models and humans. TCFs recognize specific DNA sequences through their high mobility group (HMG) domains, but invertebrate TCFs and some isoforms of vertebrate *TCF7* and *TCF7L2* contain a second DNA binding domain known as the C-clamp. This review will cover the basic properties of C-clamps and their importance in Wnt signaling, using data from *Drosophila*, *C. elegans*, and mammalian cell culture. The connection between C-clamp containing TCFs and colorectal cancer will also be discussed.

## 1. Introduction to the TCF Family

The T-cell Factor/Lymphoid Enhancer Factor (TCF/LEF; hereafter referred to as TCF) family of transcription factors are major nuclear mediators of the Wnt/β-catenin signaling pathway [1,2,3]. In *Drosophila*, depletion of the sole *TCF* gene (*TCF*/*Pangolin* or *TCF*/*Pan*) results in severe patterning defects in the embryo and larval imaginal discs, indicative of loss of Wnt signaling [4,5,6]. Similarly, in *C. elegans*, the single *TCF* gene *POP-1* has similar phenotypes to *Wnt* or *β-catenin* mutants in gonadal stem cell specification and left Q (QL) neuroblast migration [7,8], and regulates Wnt transcriptional targets in early embryogenesis [9,10,11,12]. While there is evidence that TCF is the predominant transcription factor in the Wnt pathway in invertebrates, the situation is less clear in vertebrates. No vertebrate *TCF* loss-of-function mutant has been found that resembles the gastrulation defect phenotype seen in mutants of other positive acting factors in the Wnt pathway—e.g., *Wnt3*, *β-catenin*, and *LRP5*, *LRP6* double mutants [2]. While this could be due to redundancy among different mammalian TCFs, there is also a growing list of non-TCF transcription factors (e.g., FOXO and SOX proteins and nuclear receptors) which have the ability to bind β-catenin and activate transcriptional targets [1,2,13,14]. While these complexities need to be resolved, this chapter will focus on the TCF family of proteins in Wnt-dependent gene regulation.

Unlike invertebrates, which typically have a single *TCF* ortholog, mammals have four *TCF* genes: *TCF7* (also known as *TCF1*), *LEF1*, *TCF7L1* (also known as *TCF3*), and *TCF7L2* (also known as TCF4) [1,2]. Additional diversity comes from the fact that mammalian *TCF* genes undergo extensive alternative splicing and have multiple promoters [2]. LEF1 is primarily known as a transcriptional activator of Wnt target genes [15,16], while TCF7L1 appears to act mainly as a repressor [15,17]. TCF7 and TCF7L2 can act as both activators and repressors of Wnt targets, depending on the context [15,18,19]. TCFs—particularly TCF7 and TCF7L2—have been implicated across different tumor types [20,21,22], with a particularly strong link to colorectal cancer [18,23,24,25,26].

All TCF family members share two highly-conserved domains: the β-catenin binding domain and the High Mobility Group (HMG) DNA-binding domain [2]. Originally discovered through yeast two-hybrid screens, the N-terminal β-catenin binding domain interacts with the intracellular messenger β-catenin, which translocates from the cytoplasm to the nucleus upon Wnt stimulation [6,27,28]. Deletion of this domain creates a dominant negative version of TCF, presumably by outcompeting endogenous TCFs for binding to target sites on chromatin [6,28]. Such dominant negative isoforms occur naturally for TCF7, LEF1, and TCF7L2, where they play physiologically relevant roles in antagonizing Wnt/β-catenin signaling [29,30,31,32].

The HMG domains found in TCFs belong to the HMGB subgroup of the larger HMG superfamily [33,34]. These HMG domains are comprised of three α-helices, which interact with DNA through minor groove contacts and cause a DNA bend between 90° and 130° [35,36]. An adjacent basic tail C-terminal to the third α-helix also contributes to this binding and bending [35]. Interestingly, the HMG domain of LEF1 is significantly disordered in solution, only adopting its full helical structure when complexed with DNA [37]. The HMG domains of invertebrate and vertebrate TCFs are highly conserved [1], and all family members preferentially bind a consensus in vitro defined by SCTTTGATS (S = G/C) [6,38,39]. In vivo, these sites have been found in many Wnt-responsive enhancers [1,40], and this motif is enriched in TCF7L2 and β-catenin binding peaks in chromatin immunoprecipitation experiments [41,42,43].

## 2. The C-Clamp: Biochemical Properties and Functional Roles

**Figure 1:**
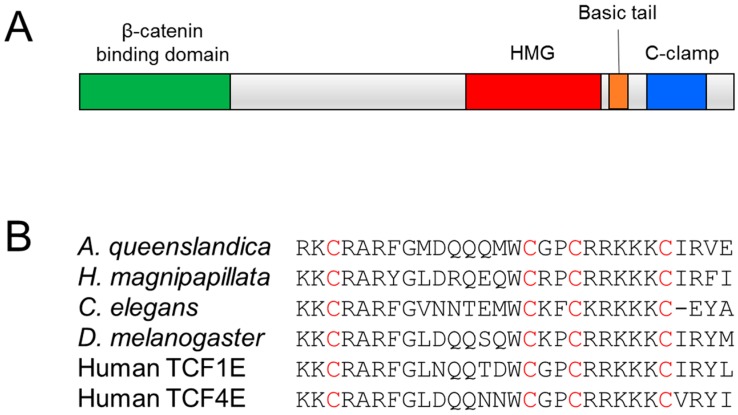
Conserved domains of the T-cell Factor/Lymphoid Enhancer Factor (TCF) family of transcription factors. (**A**) Schematic of a generic TCF, showing the β-catenin binding domain (green), the high mobility group (HMG) domain (red), the basic tail (orange), and the C-clamp (blue). The first three motifs are found in all TCFs, with the C-clamp being present in most invertebrate TCFs and in some vertebrate TCF1E and TCF4E isoforms; (**B**) Alignment of the C-clamps across metazoan TCFs. Sequences from four invertebrate TCFs and human TCF1E and TCF4E are included. Conserved cysteine residues across the 6 sequences are highlighted in red.

**Figure 2:**
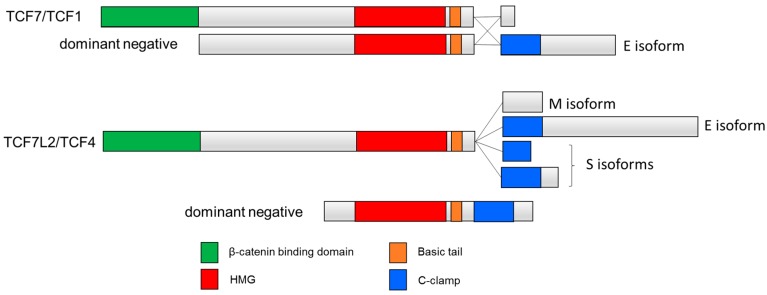
C-clamp containing human TCF isoforms. Inclusion of the C-clamp is seen in the E-tail-containing isoforms TCF1E and TCF4E. Other isoforms include M isoforms that lack the C-clamp and S isoforms that contain a truncated C-clamp [46]. Dominant negative isoforms dnTCF7 and dnTCF7L2 lack the β-catenin binding domain.

Many TCFs also contain a second DNA binding domain, termed the cysteine clamp or C-clamp, which is C-terminal to the HMG domain (Figure 1). Originally discovered by Waterman and colleagues, the C-clamp is present in most invertebrate TCFs as well as the so-called E-tail-containing isoforms of the vertebrate TCF7 and TCF7L2 [39]—hereafter referred to as TCF1E and TCF4E. In vertebrate TCFs, the E-tail is one of several alternatively spliced patterns corresponding to the C-terminal portion of TCF7 and TCF7L2 [44,45]. In addition to TCF1E and TCF4E isoforms, many other TCF7 and TCF7L2 isoforms lack the C-clamp, and some contain a truncated C-clamp [46] (Figure 2).

**Figure 3:**
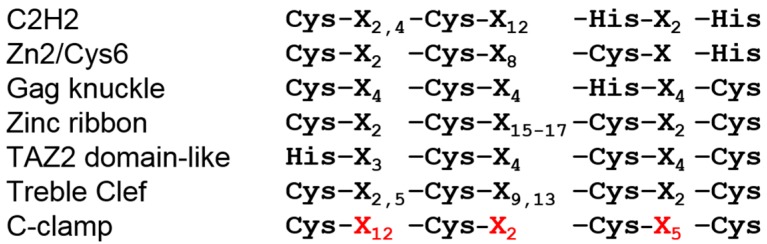
The spacing of cysteine residues in the C-clamp is unique and distinct from other zinc finger motifs. In all six zinc-finger superfamilies, the spacing between the zinc-coordinating residues (Cys or His) is even (Gag knuckle and TAZ2 domain-like) or a pattern where the first two and last two Cys/His residues are close together, separated by a larger stretch of amino acids. The C-clamp is unique in that there are twelve residues between the first and second Cys residues, and only two between the second and third. The unique number of residues between the cysteine residues in the C-clamp are highlighted in red. See text for further explanation.

The C-clamp gets its name from its hallmark of four highly-conserved cysteines (Figure 1) [39]. While the crystal structure of the C-clamp is yet to be determined, there is convincing evidence that the C-clamp forms a DNA-binding zinc finger domain. The C-clamp’s DNA binding activity requires the presence of a zinc ion, and recombinant C-clamp contains near-stoichiometric quantities of zinc [47]. In addition, mutation of any of the four cysteines results in a non-functional C-clamp [39,47,48,49]. In this way, the C-clamp is similar to the four cysteine treble clef zinc fingers found in nuclear receptors [50]. However, the spacing of the cysteine residues of the C-clamp is distinct from treble clef zinc fingers [47]. Even compared to the entire family of zinc finger motifs [51,52], the Cys-X_12_-Cys-X_2_-Cys-X_5_-Cys spacing of C-clamps is distinct from all other classes (Figure 3). In addition to the cysteines, two stretches of basic amino acid residues—the first adjacent to the first cysteine and the second between the third and fourth cysteines—contribute to both DNA binding and activation of a reporter construct in cell culture [47]. Based on the available data, one can envision that the C-clamp is a zinc-finger domain that makes direct contacts with the DNA phosphate backbone and base pairs through its basic residue stretches.

Like the HMG domain, the C-clamp can recognize DNA in a sequence-specific manner. The presence of a C-clamp in mammalian TCF1E and TCF4E allows these proteins to recognize a motif of RCCG in addition to the classic HMG binding site [39,46,53]. In *Drosophila*, the C-clamp of TCF/Pan is necessary and sufficient for binding to a GC-rich motif, referred to as the Helper site [47,48]. In flies, the Helper site consensus is GCCGCCR (R = A/G), while the slightly divergent C-clamp of *C. elegans* POP-1 has a consensus of GCCRAnW (W = A/T) [54]. Helper sites are found near functional HMG sites in more than a dozen Wnt-responsive enhancers in flies and worms, and mutation of these Helper sites abolishes activation by the Wnt pathway [48,54,55]. Helper-like RCCG motifs are found in the regulatory DNA of mammalian *LEF1* and *CDX1* genes, which require an intact C-clamp for activation by Wnt signaling [39,56]. Helper-like sites have also been found in the promoter regions of several other human Wnt targets [42,53], including genes (e.g., *SP5*, *CDX2,* and *MYC*) that are upregulated in colorectal cancer (CRC) [49,53]. Taken together, the data support a model where the DNA binding specificity of TCFs is enhanced by the presence of a C-clamp, which allows bipartite recognition via HMG domain–HMG site and C-clamp–Helper site interactions [2,55].

**Figure 4:**
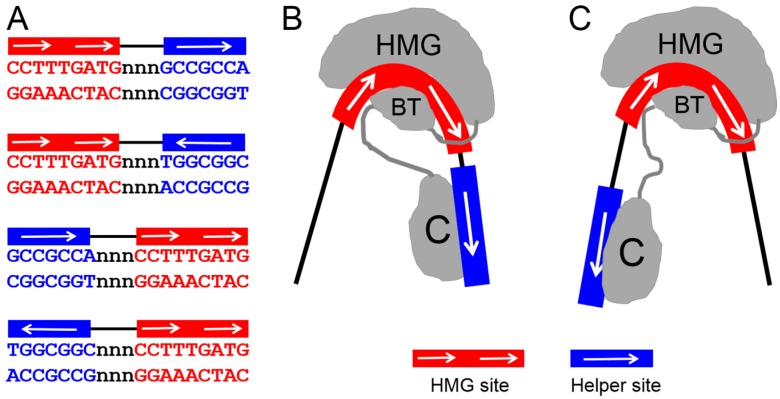
Bipartite but flexible DNA recognition model for TCF. TCF/Pan recognizes DNA through simultaneous HMG domain–HMG site and C-clamp–Helper site interactions. (**A**) Sequence of the four possible HMG–Helper site configurations. All four orientations increase binding affinity over HMG sites alone [55], and have biological activity in some cells [49,55]; (**B**,**C**) A model that can explain how a single TCF molecule can bind to all four orientations. The ability of the HMG domain to dramatically bend DNA [35] could allow the C-clamp to reach Helper sites either downstream (**B**) or upstream (**C**) of the HMG site. In addition, the semi-palindromic nature of the Helper site (**A**) can explain how either orientation can be recognized in a HMG–Helper site pair. Figure adapted from Archbold et al. [55].

In addition to enhancing DNA specificity, the architecture of HMG–Helper site pairs also contributes to more nuanced transcriptional regulation of Wnt-regulated enhancers. The spacing and orientation of these motifs were systemically examined for TCF/Pan. Some constraints were identified (i.e., two of the four possible orientations were preferentially bound by TCF/Pan), but there was also a remarkable level of flexibility in DNA recognition, with the presence of a Helper site in any orientation near a HMG site improving TCF binding and Wnt target activation [55]. This is possibly due to the ability of the HMG domain to bend DNA, which may allow the C-clamp to “swing” and interact with Helper sites located upstream or downstream of HMG sites (Figure 4). In addition, the semi-palindromic nature of Helper sites makes it difficult to unambiguously define whether a HMG–Helper pair has a tandem or inverted orientation [55]. Interestingly, the orientation of HMG–Helper site pairs in transgenic fly reporter assays had a profound effect on the tissue specificity of Wnt-dependent transcriptional activation [55]. While the mechanism of this mode of regulation remains to be determined, it is worth noting that in mammalian Wnt targets, functional Helper sites can be either upstream or downstream of their cognate HMG sites [49,53].

TCFs are thought to regulate many Wnt targets through a transcriptional switch mechanism, and there is data indicating that this regulation involves differential use of Helper sites. In this model, targets are repressed in the absence of signaling by TCF-dependent recruitment of co-repressors to Wnt target gene chromatin [3,57]. β-catenin binding to TCF inactivates or displaces co-repressors and recruits co-activators to the complex, leading to transcriptional activation. In *Drosophila* and *C. elegans*, where a single TCF is responsible for both basal repression and β-catenin-dependent activation, activation of Wnt targets requires both HMG and Helper sites [54]. While HMG sites also mediate basal repression, the C-clamp–Helper site interaction was found to be dispensable for TCF-dependent repression in the absence of Wnt signaling [54]. It is interesting to note that in vertebrates, TCF7L1, whose isoforms do not contain a C-clamp, is thought to mediate the majority of basal repression of Wnt targets [15,17,58,59,60,61].

In addition to the classic TCF transcriptional switch, another type of switch has been described in *Drosophila* hematopoietic cells for genes that are repressed by Wnt/β-catenin signaling. In these cases, TCF/Pan activates the gene’s expression in the absence of signaling, while β-catenin promotes transcriptional repression [62,63]. The TCF binding sites mediating this “reverse transcriptional switch” are distinct from classic ones, with the HMG domain site having the consensus WGAWAW for HMG sites and the Helper sites KCCSSNWW (K = G/T) [63]. These novel sites are instructive, in that a Wnt-repressed element can be converted to an activated one simply by swapping the novel HMG and Helper sites to the classic consensus, and vice versa—i.e., an activated element can be converted to a repressed element by converting the sites to the novel consensus [63]. Both the HMG and Helper sites must be swapped in order to achieve this dramatic reprogramming of the transcriptional output. These data support a model where TCF and the TCF-β-catenin complex are allosterically regulated by the specific HMG/Helper sites it associates with, influencing the subsequent recruitment of additional co-regulators [63].

It is important to note that the above-mentioned DNA binding properties of TCF have been studied primarily in invertebrates. However, the primary sequences of the HMG and C-clamps of TCF1E and TCF4E are very similar to TCF/Pan (Figure 1), suggesting that they share similar biochemical properties. While this needs to be investigated more directly, several specific Wnt target genes (many of which are associated with oncogenesis) require TCF1E and TCF4E isoforms for Wnt-dependent expression [39,46,49,53,56,64].

## 3. Other C-Clamp Containing Proteins

In addition to TCF1E and TCF4E, the C-clamp is present in three other proteins in mammalian genomes [65,66,67,68]. The best characterized are Huntington Disease Binding Proteins (HDBP) 1 and 2, both of which have been associated with Huntington’s Disease [67]. HDBP1 is also known as SLC2A4 regulator (SLC2A4RG) or GLUT4 enhancer factor (GLUT4EF) [69,70], and have been linked to increased risk of intestinal disorders [71,72]. In addition to TCF/Pan, *Drosophila* has only one other C-clamp-containing protein, known as fly Glut4EF, which regulates wing position in adult flies [73]. HDBP2—more commonly known as ZNF395—has also been implicated in various human cancers, although in conflicting roles. It’s thought to act as an oncogene, since elevated levels of this transcription factor may support cancer progression in hypoxia-induced cancers, such as glioblastomas and neuroblastomas, through the innate immune response pathway [74]. However, evidence in liver and breast cancer cell lines suggest a tumor suppressor role for ZNF395, potentially via repression of a pro-metastatic gene expression program [75,76]. The third C-clamp protein, known as ZNF704 or glucocorticoid induced gene (GIG1) [68] has recently been suggested to be a candidate for a factor influencing human longevity [77].

## 4. The Role of C-Clamp Containing TCFs in Colorectal Cancer

Elevated Wnt signaling is thought to be oncogenic in many human cancers, but perhaps the most compelling case is in CRC [78,79]. TCF7L2 is a major player in regulating Wnt transcriptional targets in this context [80]. This is supported by mouse genetics, where *TCF7L2* whole animal or conditional knockouts result in a loss of proliferative capacity of the intestinal epithelia [81,82]. In several CRC cell lines, expression of a dominant negative TCF7L2 (i.e., lacking the β-catenin binding domain) or siRNA knockdown of *TCF7L2* inhibits growth and results in apoptosis [26,83]. Consistent with a positive role in Wnt signaling, some CRCs contain a recurring *TCF7L2* fusion that presumably allows it to activate transcription in a β-catenin-independent manner [84]. 

While the short summary above reflects the prevailing view [85], it should be noted that there are also reports supporting a role for TCF7L2 repressing Wnt signaling [18] and acting as a tumor suppressor in a mouse model of CRC [23]. While these discrepancies could be explained by differences in the experimental protocols [82,83], it is also possible that TCF7L2 mediates both sides of the TCF transcriptional switch, and one or the other activity is emphasized in a given study.

TCF7 has also been implicated in CRC and intestinal biology, acting in an antagonistic manner to TCF7L2. In non-pathological conditions, intestinal cells largely express a truncated TCF7 from an intronic promoter, producing a protein lacking the N-terminal β-catenin binding domain (dnTCF7) [25,29,45]. Depletion of *TCF7* resulted in an increase in adenoma formation in the adult gut and markedly increased tumor incidence in *multiple intestinal neoplasia* (*min*) mice, which are heterozygous for a stop codon in the tumor suppressor gene APC (*Apc^min^*) [29]. Presumably, loss of dnTCF7 allows TCF7L2 to drive the Wnt pro-proliferative transcriptional program in these cells [25,29]. 

At odds with the mouse knockout data, TCF7 has also been reported to have an oncogenic role in one CRC cell line [18]. One explanation for this discrepancy comes from a study by Waterman and colleagues, who demonstrated a dramatic difference in TCF7 isoform expression and subcellular localization between normal and CRC cells [25]. While normal cells contain mostly nuclear dnTCF7, CRC cells have decreased expression of dnTCF7 and a dramatic increase in the expression of full length TCF7. This TCF7 resides largely in the cytoplasm. This subcellular localization is regulated by a Calmodulin-dependent kinase II (CamKII)-Wnt-CamKII feed-forward loop [25]. The authors suggest that this regulation can explain the dual role of TCF7 in CRC, where TCF7 is converted from a tumor suppressor to an oncogene by Wnt and CamKII signaling [25]. 

While most studies on the role of TCF7 and TCF7L2 in CRC do not indicate whether the isoform used contains a C-clamp, Waterman and colleagues have examined this issue in some detail. Originally, they noted that TCF1E and TCF4E could specifically activate reporters containing regulatory DNA from the *Lef1* and *Cdx1* promoters [56,86]. Subsequent work indicated that a functional C-clamp was required for this regulation [39,49]. Overexpression of dnTCF7 had previously been associated with stalling of the G1 to S phase transition in CRC cells, in a p21-dependent manner [26]. This effect of dnTCF7 is dependent on the presence of the C-clamp [39]. Transcriptome analysis of CRC cells expressing dnTCF7 with a functional or mutant C-clamp demonstrated that the regulation of many Wnt targets is C-clamp dependent. Several of these C-clamp-specific targets are inhibitors of p21 (e.g., *SP5*, *TGIF*, *YAP1*), suggesting a model where C-clamp containing TCF activates their expression to bypass a p21 block in the cell cycle of CRC cells [49]. Transcriptional regulation of one of these target genes, *SP5*, was found to be dependent on C-clamp–Helper site interactions, suggesting a direct role for the C-clamp in the modulation of the cell cycle in intestinal cancer cells [49]. This was extended to a genomic level by ChiP-seq experiments with TCF7 with or without the C-clamp, which support the view that many biologically important Wnt targets in CRC are C-clamp/Helper site dependent [53]. Consistent with this, most dnTCF7 in intestinal cells contains a C-clamp [25].

Another research group explored a broader role for the C-clamp across multiple cancers through transcriptional regulation of the *TMEPAI* (transmembrane prostate androgen-induced RNA) gene [87]. *TMEPAI* activation is dependent on both the TGF-β and Wnt pathways [88], and TMEPAI has been implicated in intestinal polyp formation in *Apc^Min/+^* mice [89,90], as well as in human breast cancer, colon cancer, renal cell carcinoma, and lung adenocarcinoma [91,92,93,94]. Nakano and colleagues found that only E isoforms of TCFs efficiently activated a *TMEPAI* reporter construct in the presence of TGF-β stimulation, but not isoforms lacking this domain [87]. Moreover, *TCF7L2* mutants with either a C-clamp deletion or C-clamp point mutation (C463A) were no longer able to potentiate reporter activity, indicating that the C-clamp is necessary for this regulation [87].

## 5. Final Perspective

While the importance of the C-clamp in Wnt/TCF regulation of CRC remains to be established, the existing data indicates that more attention needs to be paid to this DNA binding domain. For example, one recent report on the reciprocal regulation of TCF7L2 and the transcription factor ZEB1 in CRC progression utilized a TCF7L2 isoform containing the C-clamp, but failed to consider whether this domain played a role in the regulation of several Wnt target genes [95]. Moving forward, testing the function of TCF7L2 isoforms with and without the C-clamp in the regulation of specific targets should become the standard.

A rigorous test of the C-clamp’s importance in Wnt transcriptional regulation would be to engineer a mouse mutant where the TCF1E or TCF4E isoforms are not expressed. Deletion of the C-clamp-containing exon 9 in the *TCF*7 locus [45], and exons 14 and 15 in TCF7 [46] would generate such a double *TCF1E*, *TCF4E* knockout. *TCF7* and *TCF7L2* compound null mouse embryos exhibit severe hindgut defects, with anterior transformation of the gastrointestinal tract [96]. It is tempting to speculate that *TCF1E*, *TCF4E* mutants would display more nuanced phenotypes. If these mice had normal intestines, they could be placed in CRC mouse models such as *Apc^min^* [97], or mice carrying a *CDX2P-NLS Cre* recombinase transgene and a *loxP*-targeted *Apc* allele [98]. Crossing a *TCF1E*, *TCF4E* double mutant into either background could provide important insights into the tumor-promoting versus tumor-suppressing roles of C-clamp-containing TCF isoforms.
